# Bacterial vesicles-initiated *in-situ* spray-polymerized coating enables synergistic antibacterial-photothermal functionality for accelerating wound healing

**DOI:** 10.7150/thno.126844

**Published:** 2026-02-11

**Authors:** Dan Wang, Shuya Liang, Zhijuan Ai, Qian Kong, Dongming Xing, Zhenping Cao, Zhongmin Geng

**Affiliations:** 1 Cancer Institute, The Affiliated Hospital of Qingdao University, Qingdao University, Qingdao 266071, China.; 2 School of Basic Medicine Qingdao University, Qingdao University, Qingdao 266071, China.; 3 Department of Dermatology, The Affiliated Hospital of Qingdao University, Qingdao, China.; 4 Shanghai Key Laboratory for Nucleic Acid Chemistry and Nanomedicine, Institute of Molecular Medicine, Renji Hospital, School of Medicine, Shanghai Jiao Tong University, Shanghai 200127, China.

**Keywords:** bacterial extracellular vesicles, polypyrrole coating, antibacterial activity, photothermal responsiveness, wound healing

## Abstract

**Background:**

Although microbial therapies can address the harm to beneficial bacteria and microbiome balance caused by traditional antibacterial treatments in skin damage and infection, their pathogenic potential limits clinical application. Bacterial extracellular vesicles (BEVs) offer a safer alternative by targeting microbes and modulating immunity.

**Methods:**

*Lactobacillus reuteri*-derived BEVs (LBEVs) are functionalized with Fe**^3+^** via electrostatic adsorption, and co-sprayed with pyrrole monomers onto wounds to initiate oxidative polymerization and then form conformal polypyrrole coatings (LBEVs-PPy). Thanks to the natural antibacterial activity of LBEVs, the LBEVs-PPy coating could inhibit the growth of pathogens efficiently. Furthermore, the mild hyperthermia induced by PPy's NIR-triggered photothermal activation significantly upregulates the expression of angiogenic regulators.

**Results:**

*In vitro*, LBEVs effectively inhibited the growth of *S. aureus*, *E. coli*, and *S. epidermidis*, demonstrating potent antibacterial efficacy. Following mild hyperthermia (42 °C for 1 h), HUVECs showed elevated expression of angiogenic regulators, including *VEGFA* and *ANGPT1*. This treatment also activates HSP90/p-eNOS pathway in HUVECs, thereby accelerating angiogenesis. In a mouse model of skin damage and infection, LBEVs-PPy coating significantly accelerates wound healing through synergistic mechanisms that integrate the antibacterial activity of LBEVs and the photothermal effect of PPy.

**Conclusions:**

Our research developed an in-situ spray-polymerized coating integrating antibacterial and photothermal modalities, thus presenting a promising biotherapeutic platform for clinical wound management and tissue regeneration.

## Introduction

As the primary physical barrier of the host, the skin interface plays a significant role in defending against the invasion of microbial pathogens and a series of physical and chemical damages 1, 2. However, upon skin damage, harmful bacteria can invade viable tissues, leading to wound infections and potentially serious tissue destruction. Antimicrobial resistance represents a critical and urgent threat to global public health, already claiming approximately 700,000 lives per year. It is predicted that humanity will face over 10 million annual deaths due to bacterial infections by 2050 3. To combat bacterial infections, varieties of new antimicrobial drugs and innovative materials, such as new antibiotics, antimicrobial peptides, and antibacterial nanoparticles, have been developed 4, 5. Although current strategies effectively inhibit bacterial growth and promote wound healing, they often indiscriminately kill both pathogenic and beneficial bacteria, disrupting the skin microbiome balance 6. Accordingly, developing selective antimicrobial strategies that inhibit pathogens without compromising commensal bacteria remains a critical challenge.

In recent years, microbiome-based therapies have garnered significant attention for treating diverse diseases via bacterial interference and immune regulation 7, 8. Certain commensal bacteria and beneficial bacteria can establish unique local microenvironments via substantial secretion of metabolites and antimicrobials, fostering their own proliferation while suppressing competitors. For example, lactobacilli effectively treat urinary tract infections by restoring vaginal microbiota balance 9. However, the pathogenic and immunogenic properties of bacteria have largely limited the application of biotherapy. Bacterial extracellular vesicles (BEVs), spherical particles delimited by a lipid bilayer, have been proven to carry a diverse range of bioactive constituents (such as proteins, nucleic acids, lipids, and virulence factors) directly derived from their parent cells 10. Saroj et al. utilized mint-derived extracellular nanovesicles loaded into a hydrogel to accelerate healing of bacteria-infected wounds 11. Similarly, the hydrogel incorporating Lac-EVs (from Lactobacillus bulgaricus) effectively achieved 99.3% closure of diabetic full-thickness wounds by inducing macrophage M2 polarization and promoting angiogenesis 12. Therefore, developing a BEVs-based antimicrobial wound dressing that can effectively regulate the wound microenvironment and promote healing is highly desirable.

**Figure 1:**
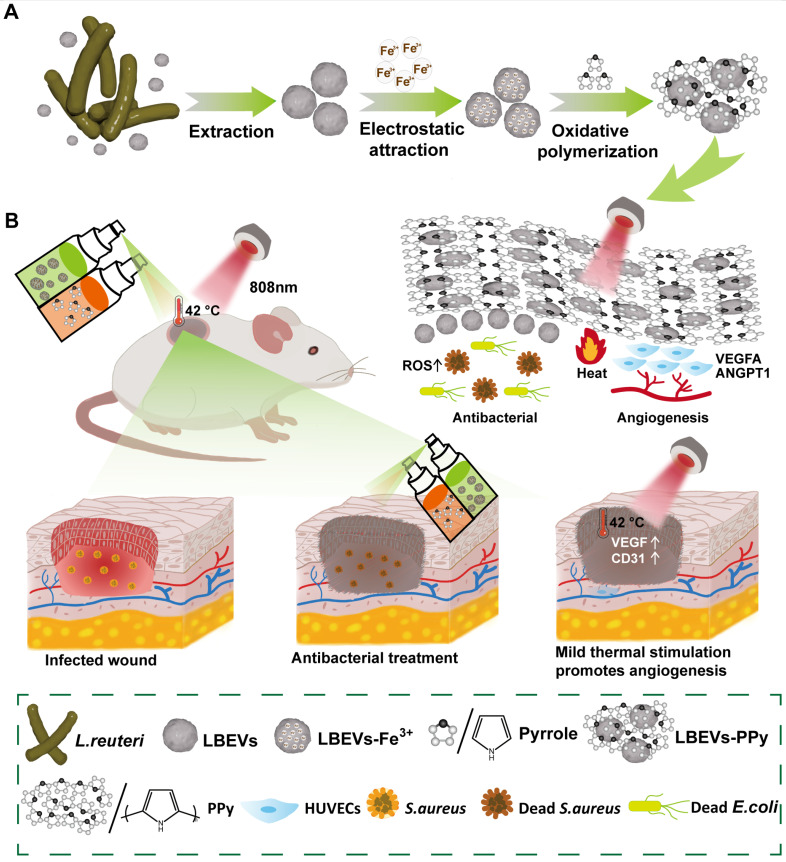
Schematic illustration of preparation and application of the LBEVs-PPy. (A) LBEVs were extracted from *L. reuteri* and then coated with Fe^3+^ to initiate in-situ spray-polymerization of LBEVs-PPy. (B) The LBEVs-PPy, with excellent antimicrobial and angiogenic capabilities, can promote the healing of infected wounds.

Impaired angiogenesis following inflammation hinders nutrient and oxygen supply to wounds, leading to delayed healing 13. Previous studies have shown that mild thermal stimulation below 48 °C enhances cell recruitment and tissue regeneration, while promoting angiogenesis and wound healing. For instance, Zhang et al. have constructed a graphene oxide (GO)-incorporated photothermal hydrogel that delivers stable mild thermal stimulation 14. This stimulation modulates immune responses and ultimately promotes wound healing. In addition, the most common ways to deliver BEVs, such as oral or injection, often fail to make them adhere effectively to the local lesion site, which consequently compromises their therapeutic efficacy. Biofilms, which are multicellular aggregates coated by extracellular polymeric substances, can effectively protect the bacterial cells and achieve strong adhesion to localized lesions 15. Inspired by these structures, we describe a BEVs-induced in-situ spray-polymerized coating with near-infrared (NIR) photothermal responsiveness for treating bacterial infections and accelerating wound healing. Firstly, we select *Lactobacillus reuteri* (*L. reuteri*), a heterofermentative probiotic, as the BEVs source, with the derived vesicles termed LBEVs. This strain suppresses the growth of certain pathogenic bacteria through secreting antibacterial agents. Given the negatively charged nature of bacterial outer membranes, Fe^3+^ cations adsorb onto LBEVs surfaces through electrostatic forces. Following the removal of unbound Fe^3+^, LBEVs and pyrrole monomers are simultaneously sprayed onto the wound. Fe^3+^-functionalized LBEVs act as oxidative initiators to induce pyrrole monomer in-situ polymerization, generating polypyrrole coating (LBEVs-PPy) on wound surfaces (Figure 1A). Unlike conventional photothermal therapy systems that rely on high temperatures (> 50 °C) for antibacterial action with potential tissue damage 16, 17, our work presents a dual-mechanism innovation. First, we precisely employ mild 42 °C photothermia to activate pro-angiogenic pathways for healing. Second, we integrate BEVs not through complex chemical modifications 18, but via a straightforward one-step electrostatic adsorption and in-situ polymerization process, which better preserves their intrinsic bioactivity. We show that LBEVs-PPy exhibits favorable biocompatibility with potent antibacterial efficacy against Staphylococcus aureus (*S. aureus*), *Escherichia coli* (*E. coli*), and *Staphylococcus epidermidis* (*S. epidermidis*). Concomitantly, photothermal effect significantly enhances the expression of angiogenic regulators treatment of human umbilical vein endothelial cells (HUVECs), including vascular endothelial growth factor (VEGFA) and angiopoietin-1 (ANGPT1). *In vivo* studies confirmed that accelerated wound healing by LBEVs-PPy through synergistic dual-action LBEVs-enabled antibacterial clearance coupled with PPy-photothermal angiogenesis potentiation (Figure 1B). Therefore, we believe this strategy will open new avenues for biotherapy as a safer therapeutic modality, facilitating its use for infected wound treatment and tissue repair.

## Materials and Methods

### LBEVs extraction

LBEVs were prepared according to the previous report with some modifications 19, 20. *L. reuteri* was cultivated de Man, Rogosa and Sharpe (MRS) medium at 37 °C with agitation (150 rpm) for 48 h. First, pre-cool the centrifuge to 4 °C. Then, aliquot the bacterial culture in a biosafety cabinet and place it into the centrifuge. Centrifuge at 10,000 × g for 30 min to collect the supernatant. Filter through a 0.45 µm Millipore filter membrane to remove residual bacteria, cell debris, and large particles; Pass through the 0.22 µm Millipore filter membrane again. Collect LBEVs, under 4 °C, 150,000 × g, 1.5 h, then dispersed in 0.9% sodium chloride, 4 °C under short-term preservation; -80 °C long-term preservation. Strict aseptic technique was employed to avoid contamination by extraneous microorganisms.

### Characterization of LBEVs

The average size and zeta potential of LBEVs were measured by dynamic light scattering (DLS). The total protein concentration of concentrated LBEVs was determined by the bicinchoninic acid (BCA) method. For transmission electron microscopy (TEM) imaging, samples were loaded onto Formvar carbon film-coated copper grids and then photographed. The BEVs of the *E. coli* strain with mCherry fluorescent-labeled can be observed directly by the laser scanning confocal microscopy (LSCM).

### The cultivation of *S. aureus*

Retrieve the cryopreserved *S. aureus* from storage at -80 °C. Thaw the vial rapidly in a 37 °C water bath until the contents are fully liquefied. Use a sterile pipette to transfer a small aliquot of the thawed bacterial suspension into a sterile tryptic soy broth (TSB) medium. Incubate the inoculated broth at 37 °C with constant agitation at 150 rpm.

### Antimicrobial properties of LBEVs

LBEVs at specific concentrations (BCA measured concentrations of 1, 1.5, and 2 mg/mL) Were mixed with *S. aureus* at a fixed inoculum volume of 10 µL from a stock suspension of 10^7^ CFU/mL. The mixtures were incubated for 6 h at 37 °C. The count of surviving bacteria in each group was counted using plate counting to evaluate the antimicrobial activity. *S. aureus* was stained using a Live/Dead bacterial viability kit and imaged with LSCM, while the morphology of *S. aureus* was examined using scanning electron microscopy (SEM). Furthermore, *E. coli* and *S. epidermidis* were also mixed with LBEVs (BCA measured concentrations of 2 mg/mL), and then the mixtures were serially diluted and spread onto agar plates.

### The cultivation of cells

HaCaT (human immortalized keratinocyte cell line), HUVECs, and NIH3T3 (mouse embryonic fibroblast cell line) were all cultured in DMEM medium.

### Preparation of LBEVs-Fe^3+^

The obtained LBEVs were mixed with 0.1 mol/L FeCl_3_ and incubated at room temperature for 30 min. The mixture was then subjected to ultrafast centrifugation at 150,000 × g at 4 ℃ for 1.5 h to separate the target components.

### Preparation of LBEVs-PPy

The obtained LBEVs-Fe³⁺ were mixed with pyrrole solution and allowed to react for 10 min. Subsequently, the mixture was ultracentrifuged at 150,000 × g for 1.5 h at 4 °C. The resulting LBEVs-PPy were washed with 0.9% sodium chloride and collected by repeating the centrifugation under the same conditions.

### Cell live/dead staining

LBEVs-PPy were added into the cell culture dish for photothermal treatment (42 °C, 1 h). Cell viability staining was carried out 24 h later. Specifically, after discarding the supernatant and washing three times with PBS, add 200 μL of the staining working solution to the six-well plate to cover the cells at 37 °C for 15 min. Finally, the cells were observed under fluorescence microscopy.

### Fourier transform infrared spectroscopy (FT-IR)

The LBEVs, polypyrrole (PPy), and LBEVs-PPy were freeze-dried into a powder for infrared spectroscopy analysis.

### Quantitative real-time polymerase chain reaction (qPCR)

HUVECs underwent photothermal treatment at 42 °C for 1 h in advance. Briefly, HUVECs were co-incubated with LBEVs-PPy and then exposed to 808 nm laser irradiation. The cells were harvested 12 h later. First, total RNA was extracted from the cells. First, RNA-easy reagent was added to cover the cells for easy lysis. The RNA-containing supernatant was collected by centrifugation. The same volume of isopropyl alcohol was added to precipitate RNA, and the RNA was obtained by centrifugation. Then, the RNA concentration was determined by resuspension in RNase-free ddH2O. Next, a reverse transcription system was prepared to synthesize cDNA. Finally, qPCR was performed using the obtained cDNA as the template.

### Western blot (WB)

HUVECs were seeded in a 24-well plate and cultured for 24 h. After treatment with LBEVs-PPy and photothermal stimulation at 42 °C for 1 h, cells were harvested at 6, 9, and 12 h post-treatment. And then cells were subjected to Western blot analysis for proteins HSP90 and p-eNOS.

### *In vivo* wound healing experiments

The animal experiment plan was reviewed and approved by the Animal Welfare and Ethics Committee of Qingdao University. Female 6-week-old BALB/c mice were randomly divided into four groups: no treatment (control), LBEVs group, PPy-mediated photothermal therapy group (PPy), and LBEVs-induced in-situ spray polymerization of PPy synergistic antibacterial and photothermal therapy group (LBEVs-PPy) with photothermal therapy initiated approximately 10 min post-spraying. Cyclophosphamide (100 mg/kg) was administered via intraperitoneal injection once daily on days -4 and -1 relative to the experiment initiation. Based on previous literature 21, 22, cyclophosphamide-induced immunosuppression was employed in this study to establish a clinically relevant immunocompromised state, permitting the development of a reproducible, localized polymicrobial wound infection without directly impairing the fundamental wound healing physiology. First, the mice were anesthetized, and the dorsal hair was shaved. A full-thickness skin defect model (7-mm diameter) was created on the dorsal region to simulate the wound of a skin graft donor site. To establish the infection model, each wound was inoculated with 10 μL of *S. aureus* suspension at a concentration of 1 × 10⁹ CFU/mL. Antimicrobial treatment was administered to the LBEVs and LBEVs-PPy groups by spraying microvesicles (LBEVs) at 0, 6, 12, and 24 h post-wounding. On days 7, 8, and 9 post-initiation, groups PPy and LBEVs-PPy underwent daily photothermal therapy (0.8 W/cm²) at 42 °C for 1 h. Wound areas were photographed on days 0, 3, 5, 7, 9, 11, and 13. On day 13, mice were euthanized by cervical dislocation. Full-thickness wounds with surrounding tissues were excised and processed for hematoxylin and eosin (staining) (H&E). Immunofluorescence (IF) was performed for platelet endothelial cell adhesion molecule (CD31), while immunohistochemistry detected VEGF. And then important tissues and blood were removed for analysis. Histological evaluation of liver, spleen, and kidney by H&E staining.

### Statistical analysis

All data are displayed as means ± standard error of the mean (SEM). Statistical analysis is conducted using Prism 8.0 (GraphPad, USA). Unpaired two-tailed Student's t-test is performed for comparison between two groups. A variance similarity test (F-test) is used before the t-test. Differences are considered statistically significant if p < 0.05 (*p < 0.05, **p < 0.01, ***p < 0.001, and ****p < 0.0001).

## Results and Discussion

### Characterization of the LBEVs and LBEVs-PPy

**Figure 2:**
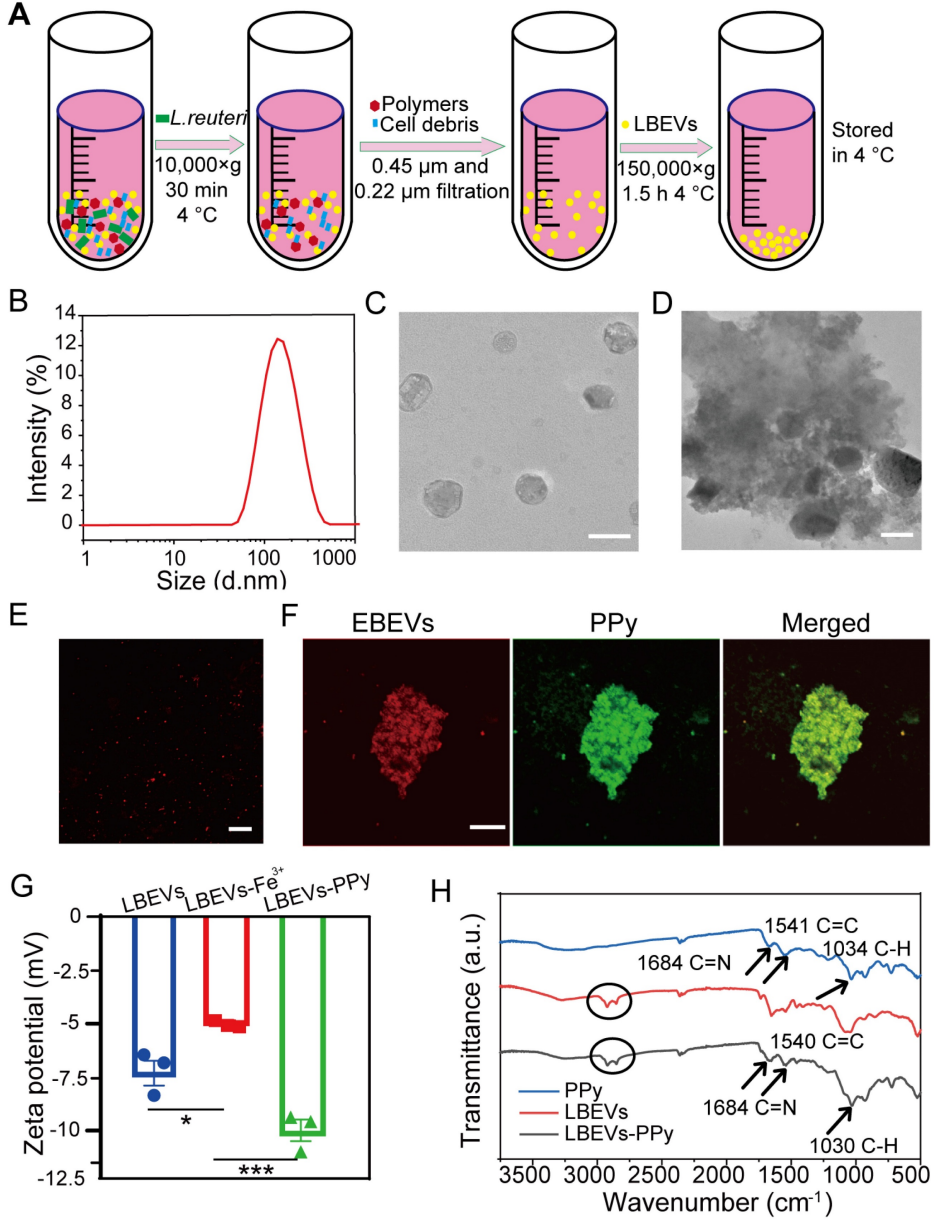
The characterization of the BEVs and BEVs-PPy. (A) Schematic diagram of LBEVs extraction. (B) Particle size distribution charts of LBEVs measured by DLS. (C) TEM images of LBEVs. Scale bar: 200 nm. (D) TEM images of LBEVs-PPy. Scale bar: 200 nm. (E) Confocal microscopic imaging of EBEVs. Scale bar: 10 μm. (F) Confocal microscopic imaging of EBEVs-PPy. Scale bar: 10 μm. (G) The zeta potential of LBEVs, LBEVs-Fe3+, and LBEVs-PPy. (H) FT-IR spectra of LBEVs, PPy, and LBEVs-PPy.

*L. reuteri*-derived extracellular vesicles were employed as model BEVs to fabricate LBEVs-PPy. As outlined in the methods, LBEVs were isolated and purified from the culture medium via ultracentrifugation (Figure 2A). Figure 2B-C illustrated that the harvested LBEVs displayed spherical morphology with predominant size distribution around 155.6 ± 6.73 nm. LBEVs concentration was quantified using the BCA protein assay, with total protein amount serving as the quantitative standard for subsequent experiments. As quantified by nanoparticle tracking analysis (NTA), the LBEVs, with a protein content of 2 mg/mL (BCA assay), exhibited a high particle concentration of 8.8 × 10¹² particles/mL (Figure S1A). The obtained LBEVs were mixed with a 0.1 mol/L FeCl_3_, and electrostatic attraction facilitated iron ions loading to yield LBEVs-Fe^3+^. To demonstrate pyrrole oxidation by iron ions on LBEVs- Fe^3+^, pyrrole monomers were mixed with LBEVs- Fe^3+^. TEM images of LBEVs-PPy revealed spherical vesicles were embedded in the PPy coating (Figure 2D). Using the same method as for LBEVs-PPy, we formulated EBEVs-PPy coatings with EBEVs isolated from mCherry-expressing *E. coli*. As shown in Figure 2E, the red fluorescence confirmed the successful preparation of EBEVs. Subsequently, EBEVs-PPy coatings were fabricated and exhibited green fluorescence that colocalized with the red-fluorescent BEVs (Figure 2F). We therefore attributed the green fluorescence to PPy aggregates synthesized on EBEVs, demonstrating effective EBEVs-PPy synthesis. To provide more objective evidence for the successful synthesis of LBEVs-PPy coating, the changes of zeta potential were measured by DLS. After mixing with FeCl_3_ solution, the zeta potential of LBEVs increased significantly from -7.2 ± 1.0 mV to -4.8 ± 0.1 mV, which is attributed to the adsorption of Fe^3+^ ions neutralizing surface negative charges (Figure 2G). Following surface polymerization, the zeta potential further decreased to -10.1 ± 0.9 mV, demonstrating PPy coating structure formation. Furthermore, the X-ray photoelectron spectroscopy analysis of LBEVs-Fe^3+^ reveals the presence of Fe^3+^. As showed in Figure S1B, the Fe2p spectrum exhibits characteristic peaks at binding energies of approximately 711.3 eV (Fe 2p_3/2_). The observed peak position is typical of Fe^3+^, as documented in previous studies. Moreover, to further confirm LBEVs-PPy coating formation, FTIR spectroscopy was employed. As indicated in Figure 2H, LBEVs-PPy exhibits characteristic PPy peaks. The peaks at 1540 cm^-1^correspond to C = C stretching, while those at 1684 cm^-1^ and 1030 cm^-1^ represent the C = N and C-H bonds, respectively. These peaks are largely consistent with literature reports 23. Additionally, peaks between 2750-3000 cm^-1^ in LBEVs-PPy may correspond to vesicle components.

### Antimicrobial properties of LBEVs

**Figure 3:**
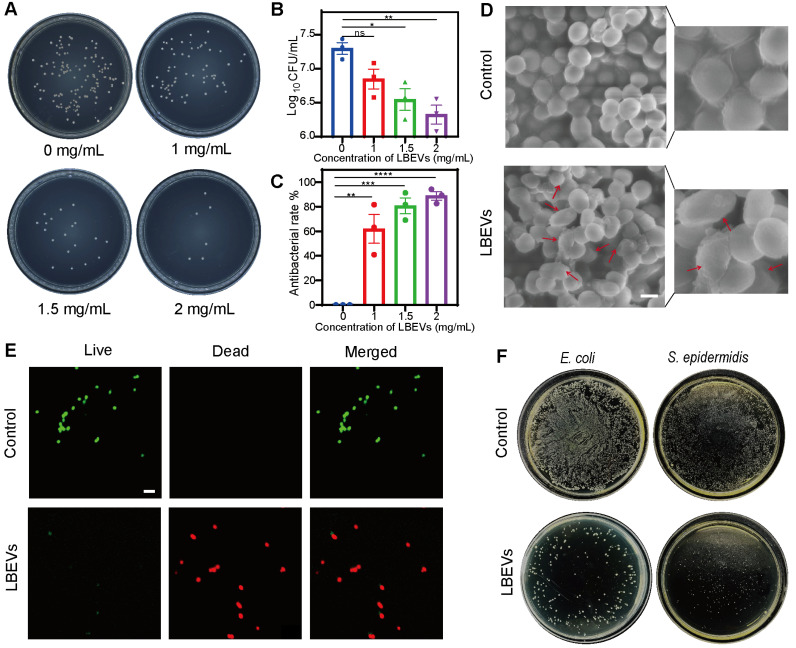
*In vitro* antimicrobial properties of LBEVs. (A) Representative plate of S.aureus co-incubated with LBEVs for 6 h at 37 °C. (B) Numbers of S.aureus after co-incubated with different LBEVs concentrations for 6 h at 37 °C. The viable bacteria were determined by colony-forming unit (CFU) counts. Data are presented as log10 (CFU/mL). *p < 0.05, **p < 0.01, ns: no significance. (C) Antibacterial rate of LBEVs. **p < 0.01, ***p < 0.001, ****p < 0.0001. Sample size n = 3 for all experiments. (D) SEM images of the morphologies of S. aureus after co-incubated with LBEVs for 6 h at 37 °C. Scale bar: 1 μm. (E) Typical CLSM images of S. aureus after co-incubation with LBEVs for 6 h at 37 °C. Bacterial cells were co-stained with calcein AM and PI before imaging. Scale bar: 5 μm. (F) Digital images of the colonies on agar plates after incubating *E. coli* and* S. epidermidis* with LBEVs. Sample size n = 3 for all experiments by a one-way ANOVA. Data are presented as means ± SEM.

*L. reuteri*, a probiotic naturally occurring in humans and animals, offers protection against the adverse effects of some harmful microorganisms and modulates immune responses 24. As reported, *L. reuteri* makes several kinds of metabolites like reuterin and reutericyclin. These substances show antibacterial activity against gram-positive and gram-negative bacteria, and help keep the balance of the skin microbial environment 25. Based on prior reports that LBEVs carry the antimicrobial metabolite reuterin (3-HPA), we sought to verify its presence in our samples 20. We performed liquid chromatography-mass spectrometry (LC-MS) analysis, which confirmed the presence of 3-HPA in the LBEVs, as shown in Figure S2A. To evaluate the antibacterial activity of the prepared LBEVs, we employed the plate-counting method. *S. aureus* was co-incubated with varying concentrations of LBEVs for 6 h, and bacterial viability was assessed based on colony-forming units (CFUs) on agar plates after the incubation period (Figure 3A). Figure 3B indicated that LBEVs had a dose-dependent antibacterial effect, suggesting that as the concentration of LBEVs increases, bacterial survival decreases.

The growth of *S. aureus* was largely suppressed when it was incubated in the presence of 1 mg/mL LBEVs in TSB medium in 6 h. It was found that the inhibition rate had almost reached 90% when the concentration of the LBEVs was raised to 2 mg/mL (Figure 3C). The long-term antibacterial activity of the LBEVs was measured by determining the bacterial survival using the time-kill-kinetics during 16-h (Figure S2b). In comparison with the control group, the culture treated with LBEVs exhibited strong growth inhibition with a growth curve that remained virtually flat throughout the experiment. Moreover, a comparative study of the antibacterial performance of PPy, Fe^3+^, LBEVs, and LBEVs -PPy as *in vitro* has been done. Under standardized *in vitro* conditions (PBS, pH 7.4), the cumulative release concentration of Fe^3+^ from the LBEVs-Fe^3+^ over 48 h was only 4.8 µM (Figure S2C). This concentration is substantially lower than the effective instantaneous concentration of Fe^3+^ (10 µM) reported by Levin *et al.* to directly induce cytotoxicity 26. The data also showed that both PPy and Fe³⁺ exhibited no appreciable antibacterial effects, same to the control. In contrast, both LBEVs and LBEVs-PPy demonstrated a considerable bacterial viability against *S. aureus*, which confirms that the antimicrobial efficacy is attributable to LBEVs (Figure S2D). Subsequently, *S. aureus* was evenly spread on agar plates and then placed the LBEVs-PPy on the plates. As shown in the Figure S2E, the inhibition zones formed obviously in the plates.

Furthermore, the SEM revealed that untreated *S. aureus* cells exhibited regular spherical morphology with smooth surfaces. In contrast, ruptured cellular membranes were observed in the group of bacteria co-incubated with LBEVs (Figure 3D). To further explore the antibacterial behavior of LBEVs, Figure 3E showed typical fluorescent images of *S. aureus*. The untreated *S. aureus* was almost alive (green-stained), with a negligible number of dead bacteria (red-stained). Conversely, since propidium iodide exclusively enters membrane-compromised cells, the dominant red fluorescence in LBEVs-treated samples demonstrated *S. aureus* membrane damage. These results confirmed effective bactericidal efficacy against the pathogen. Moreover, the antibacterial ability of LBEVs was assessed *in vitro* against *E. coli* (Gram-negative) and *S. epidermidis* (Gram-positive). As exhibited in Figure 3F and Figure S2F, relative to untreated controls of *E. coli* and *S. epidermidis*, the LBEVs treatment resulted in a marked reduction in bacterial viability, killing most bacterial cells and showing potent broad-spectrum antibacterial efficacy. Subsequently, we have explored the antibacterial mechanism of LBEVs. Studies have indicated that reuterin exerts its antibacterial effects by inducing oxidative stress 27, 28. We studied the effect of LBEVs on intracellular reactive oxygen species (ROS) levels of *E. coli* and *S. aureus* using 2',7'-Dichlorodihydrofluorescein diacetate (DCFH-DA) to evaluate the antibacterial mechanism of LBEVs. We co-incubated LBEVs with *E. coli* and *S. aureus* for 2 and 4 h. The results showed that the ROS levels increased significantly in *E. coli* after both 2 and 4 h of treatment (Figure S2G), while an increase in ROS was observed in *S. aureus* only after 4 h of incubation (Figure S2H). This confirms that the induction of oxidative stress is one of the antimicrobial mechanisms of LBEVs, which is consistent with previous reports.

### The photothermal performance of LBEVs-PPy

**Figure 4:**
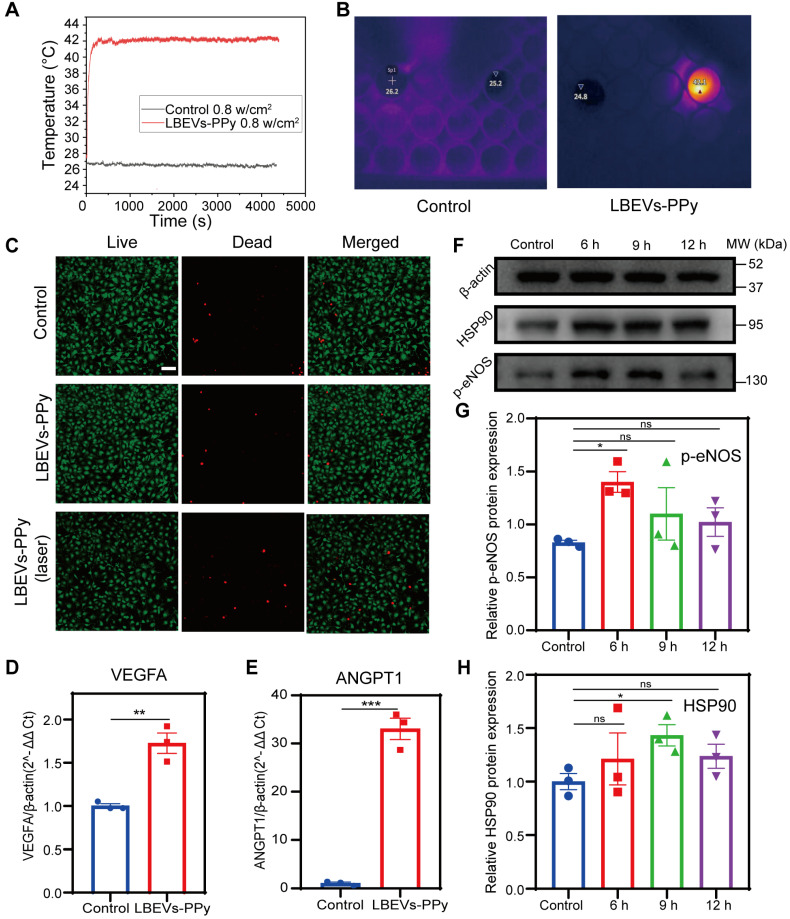
The promotion of angiogenesis by the photothermal performance of LBEVs-PPy. (A) Photothermal heating curves of LBEVs-PPy under 808 nm NIR irradiation. (B) Real-time infrared thermal images via 808 nm NIR irradiation. (C) Fluorescence microscope images of HUVECs after co-incubation with LBEVs-PPy and LBEVs-PPy + laser. Cells were co-stained with calcein AM and PI before imaging. Scale bar: 100 μm. (D) VEGFA gene expression of HUVECs after co-incubation with LBEVs-PPy under 808 nm NIR irradiation for 1 h. **p < 0.01. (E) ANGPT1 gene expression of HUVECs after co-incubation with LBEVs-PPy under 808 nm NIR irradiation for 1 h. ***p < 0.001. (F) HSP90 and p-eNOS protein expression of HUVECs after co-incubation with LBEVs-PPy under 808 nm NIR irradiation. (G) The quantitative analysis of HSP90 protein expression. *p < 0.05, ns: no significance. (H) The quantitative analysis of p-eNOS protein expression. *p < 0.05, ns: no significance. Sample size n = 3 for all experiments. Statistical significance for Figures (D) and (E) was determined using Student's t-test. For Figures (G) and (H), statistical significance was assessed using one-way ANOVA. Data are presented as means ± SEM.

PPy, an excellent biocompatibility polymer, absorbs NIR light and converts it into thermal energy, exhibiting high photothermal conversion efficiency, chemical and environmental stability, ideal for therapeutic applications 29, 30. Therefore, PPy was selected over other traditional photothermal materials such as graphene oxide, which faces uncertainties in long-term biosafety 31, 32, and polydopamine, which has relatively weaker photothermal absorption efficiency 33. To validate the photothermal efficacy of the resulting LBEVs-PPy coating, we conducted irradiation experiments using an 808 nm NIR laser at 0.8 W/cm². Under the same culture conditions, cells were split into two groups: the control group (no treatment) and the experimental group (treated with LBEVs-PPy), and both were kept in the same volume of culture. Under identical irradiation conditions (808 nm laser, 0.8 W/cm² for 1 h), a 200 µL sample was irradiated with a laser spot having an original diameter of 0.4 mm. The temperature of the control group remained nearly unchanged, whereas the LBEVs-PPy-treated group exhibited exceptional thermal conversion efficiency. The temperature of the experimental group increased rapidly upon irradiation, reaching 42 °C and maintaining for 1 h (Figure 4A). The temperature change was captured by real-time infrared thermal imaging (Figure 4B). We have included a diagram of the experimental setup as shown in the Figure S3A. To assess the durability and performance of LBEVs-PPy, we performed a long-term cycling test. Specifically, after the LBEVs-PPy coating was co-incubated with HUVECs for 72 h, it was subjected to cyclic NIR irradiation. The results clearly show that the coating retained its ability to consistently and reproducibly elevate temperature to 42 °C over multiple cycles (Figure S3B).

### Biocompatibility and bioregulation of LBEVs-PPy *in vitro*

We next evaluated the* in vitro* biocompatibility of LBEVs-PPy coatings. To assess material and hyperthermia safety profiles, cell viability assays were performed on three skin-relevant cell lines: HaCaT, HUVECs, and NIH/3T3. As shown in Figure 4C and Figure S3C-D, both the group co-cultured with LBEVs-PPy alone and the group treated with LBEVs-PPy plus 42 °C photothermal treatment for 1 h exhibited cell mortality rates comparable to the blank control group, all within acceptable thresholds. Furthermore, to avoid the potential influence of NIR irradiation, we included a laser-alone treatment group. The results indicated that the "laser-only" had negligible effects on the cells (Figure S3E). These findings demonstrated the favorable biocompatibility of the LBEVs-PPy coating and its synergistic photothermal treatment. Numerous studies have demonstrated that mild thermal treatment can promote angiogenesis and thereby accelerate wound healing 34, 35. Although reported temperatures and exposure time vary across studies, most studies maintain temperatures below 48 °C. Here, HUVECs were mildly thermally stimulated (42 °C) for 1 h, and the angiogenic factor expression was measured. Figures 4D and 4E showed that after the photothermal treatment, the expressions of *VEGFA* and *ANGPT1* genes increased significantly. Nevertheless, the *VEGFA* and *ANGPT1* genes were not expressed differently when they were treated with LBEVs alone (Figure S3F). In order to clarify the mechanism of the photothermally stimulated angiogenesis, HUVECs were harvested at a specific time after photothermal stimulation (6/9/12H). WB analysis showed that mild thermal stimulation greatly enhanced the level of heat shock protein 90 (HSP90) and phosphorylated endothelial nitric oxide synthase (p-eNOS) (Figure 4F). HSP90 protein not only has a protective effect against heat but also acts as an important molecular chaperone, regulating the expression of angiogenic molecules such as eNOS, which plays a beneficial role in angiogenesis 36, 37. p-eNOS generates large amounts of nitric oxide, promoting the proliferation and migration of endothelial cells and leading to increased vascular permeability 38. Additionally, quantitative analysis of two parameters was performed (Figure 4G-H). These results suggested that thermal stimulation may stimulates angiogenesis via VEGF, ANGPT1, and HSP90/p-eNOS pathway, as shown in Figure S3G.

### LBEVs-PPy promoting wound healing *in vivo*

**Figure 5:**
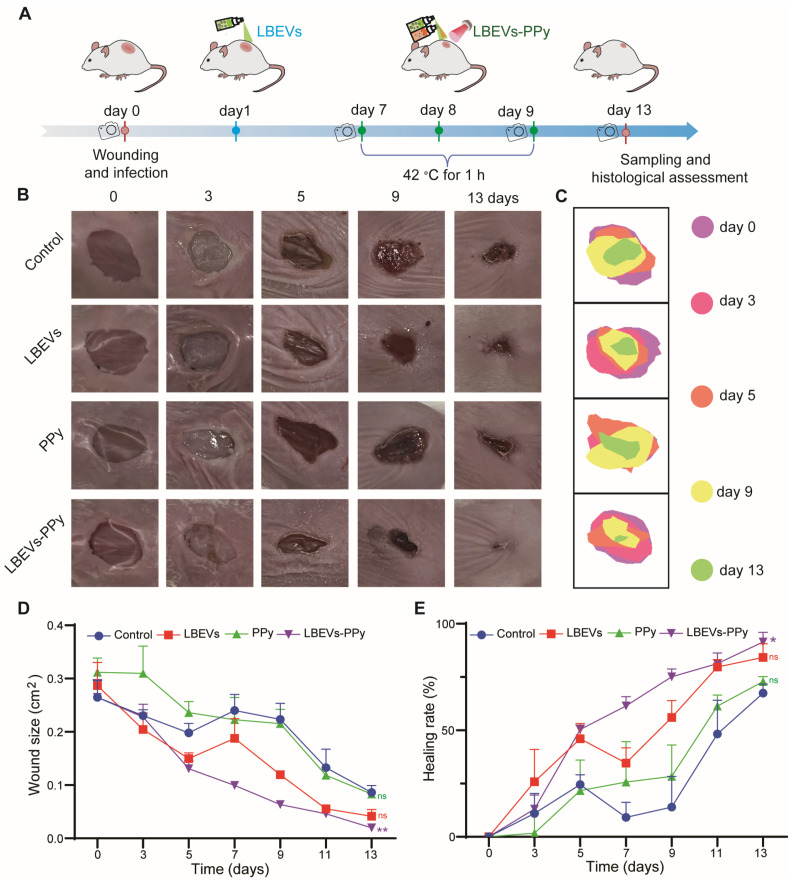
LBEVs-PPy empowered wound healing in the mouse skin wound model. (A) Schematic diagram of the wound management process. Representative wound images (B) and wound healing maps (C) of wounds with different treatments on days 0, 3, 5, 9, and 13. Quantification of (D) wound closure size and (E) wound healing rate of different groups during treatment. Sample size n = 3 for all experiments by a one-way ANOVA followed by Tukey's honest significant difference (HSD) post hoc test. Data are presented as means ± SEM. *p < 0.05, ns: no significance.

Encouraged by the *in vitro* results, we next evaluated the* in vivo* antibacterial and wound healing-promoting abilities of LBEVs-PPy in a mouse *S. aureus*-infected wound model. As shown in Figure 5A, circular full-layer skin wounds, approximately 7 mm in diameter, were created on the dorsal surface of BALB/c mice. Following wound creation, these wounds were infected with 10 µL of *S. aureus* at a concentration of 10^9^ CFU/mL. We randomly split the mice into four groups, including no treatment (control), LBEVs group (LBEVs), PPy-mediated photothermal therapy group (PPy), and LBEVs-induced *in-situ* spray polymerization of PPy synergistic antibacterial and photothermal therapy group (LBEVs-PPy).

We monitored the topographic changes of the wound healing processes by capturing digital images every 2 days. As shown in Figure 5B-C, while the wound area showed progressive reduction during the study, the rate of closure was distinctly different among treatment groups. Specifically, on the third day after surgery, both the control group and the PPy group showed obvious pus formation at and around the wound site, indicating the presence of bacterial infection. In contrast, the LBEVs and LBEVs-PPy groups showed minimal pus formation at the same time, confirming the excellent antibacterial ability of LBEVs. In addition, mice treated with LBEVs or LBEVs-PPy coatings showed accelerated wound healing by day 5, indicating successful control of *S. aureus* infection. By day 9, after photothermal therapy, the wound area in the LBEVs-PPy group significantly decreased from 26.5 ± 5.7 mm² to only 6.4 ± 0.9 mm², achieving a healing rate of 75%. In contrast, wound areas in the control, LBEVs, and PPy groups remained at 22.3 ± 5.2 mm², 2.3 ± 5.2 mm², and 21.6 ± 4.6 mm², respectively, corresponding to healing rates of only 14%, 56%, and 28%. After 13 days, wounds treated with LBEVs-PPy coating were almost completely closed and covered with new epidermis. In contrast, 33%, 16%, and 27% of wounds remained unhealed in the control, LBEVs, and PPy groups, respectively (Figure 5D-E). These findings indicated that LBEVs-PPy coating significantly promotes wound healing combining the antibacterial activity and the photothermal effect. To further investigate the effect of PPy without photothermal stimulation, we examined the specific contribution of the Fe³⁺/pyrrole. As shown in Figure S4A, after treatment with the Fe³⁺/pyrrole on day 3, pus formation was evident at the wound site. The wound closure size and healing rate showed no difference from the untreated group (Figure S4B-C).

**Figure 6:**
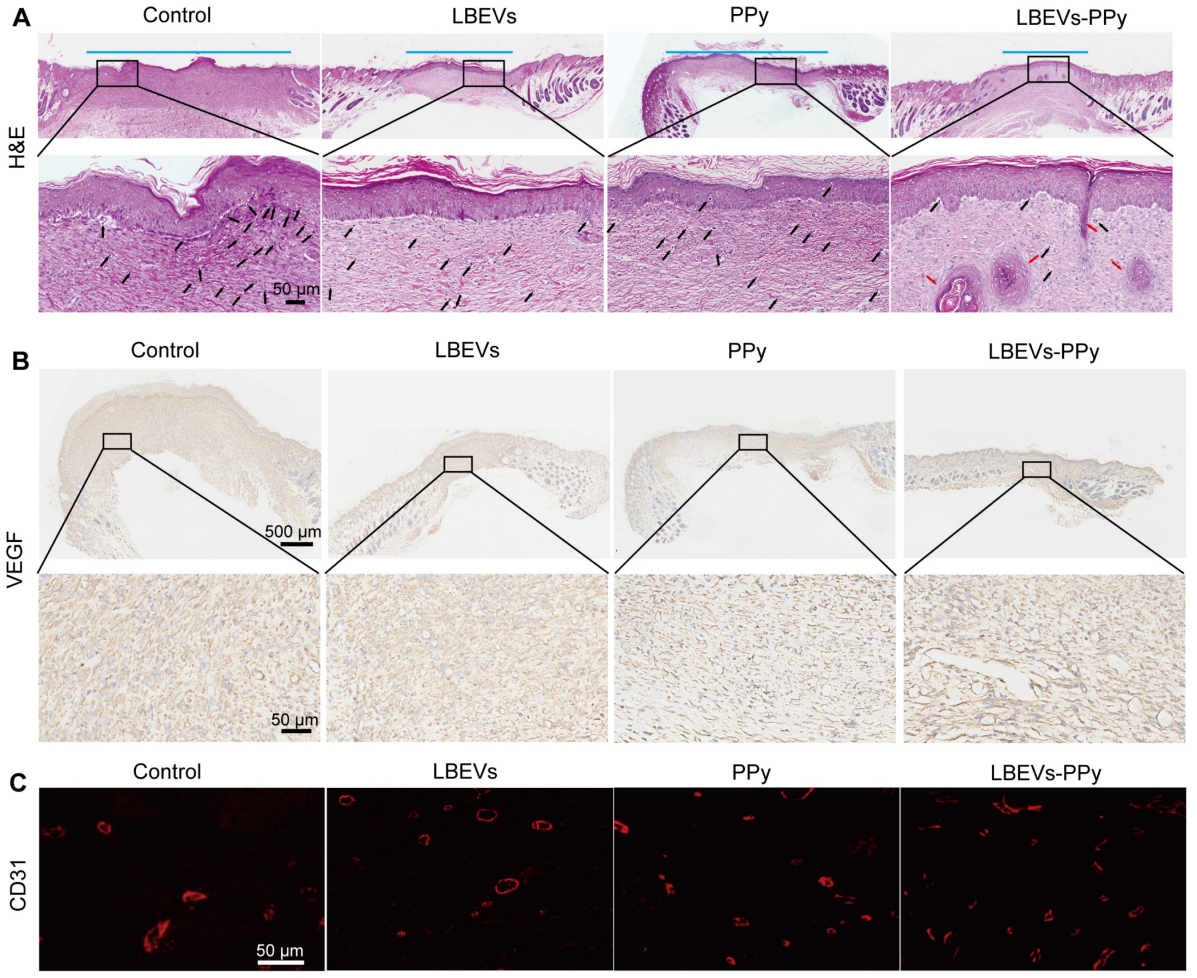
Histological images of wound tissues. (A) Representative H&E staining images of wound tissues on day 13. The blue line segments indicate the width of the wound. The black arrows denote the inflammatory cells, and the red arrows denote the hair follicles. Scale bar: 50 μm. Representative immunohistochemical staining images of VEGF (B) and immunofluorescence staining images of CD31 (C) in wound tissues on day 13. Scale bars: 500 μm, 50 μm.

To further assess wound healing progress, we performed H&E staining on wound tissues from the control, LBEVs, PPy, Fe³⁺/pyrrole, and LBEVs-PPy coating treatment groups on post-operative day 13 (Figure 6A and S4D). The control, PPy, Fe³⁺/pyrrole, and LBEVs groups showed obvious inflammation, indicated by immune cell infiltration, likely because of inadequate antibacterial efficacy and non-healing wounds. On the contrary, the LBEVs-PPy group demonstrated minimal neutrophil presence, more granulation tissue regeneration, and a certain density of hair follicles, which is attributable to the potent antibacterial activity of LBEVs and the potential of photothermal angiogenesis by PPy, thereby accelerating wound healing (representative higher-magnification images are provided in Figure S4E for better observation of inflammatory cells and hair follicle generation). Notably, significant neovascularization and epidermal regeneration in the LBEVs-PPy group revealed accelerated wound healing through photothermal therapy. Previous research confirms that angiogenesis subsequent to inflammation resolution promotes wound healing processes by supplying growth factors and nutrients that drive fibroblast activity, collagen synthesis, and re-epithelialization 39. The growth of new blood vessels was evaluated by performing immunohistochemical staining for the angiogenic growth factor VEGF. As shown in Figure 6B, low VEGF expression was observed in control, LBEVs, and PPy groups, whereas the LBEVs-PPy group exhibited the highest VEGF levels among all groups; subsequent quantitative analysis (Figure S4F) confirmed a significant increase in VEGF expression in the LBEVs-PPy group compared to both the control and LBEVs groups. Accordingly, the results of immunofluorescence staining revealed that on the 13 th day after surgery, the expression of CD31 (a common angiogenic marker) in the LBEVs-PPy group was significantly elevated and exceeded that of the control, LBEVs, and PPy groups, indicating the increased angiogenic activity (Figure 6C). On the whole, all these data proved the effectiveness of the LBEVs-PPy coating as a bacterial infection inhibitor and accelerated wound healing.

**Figure 7:**
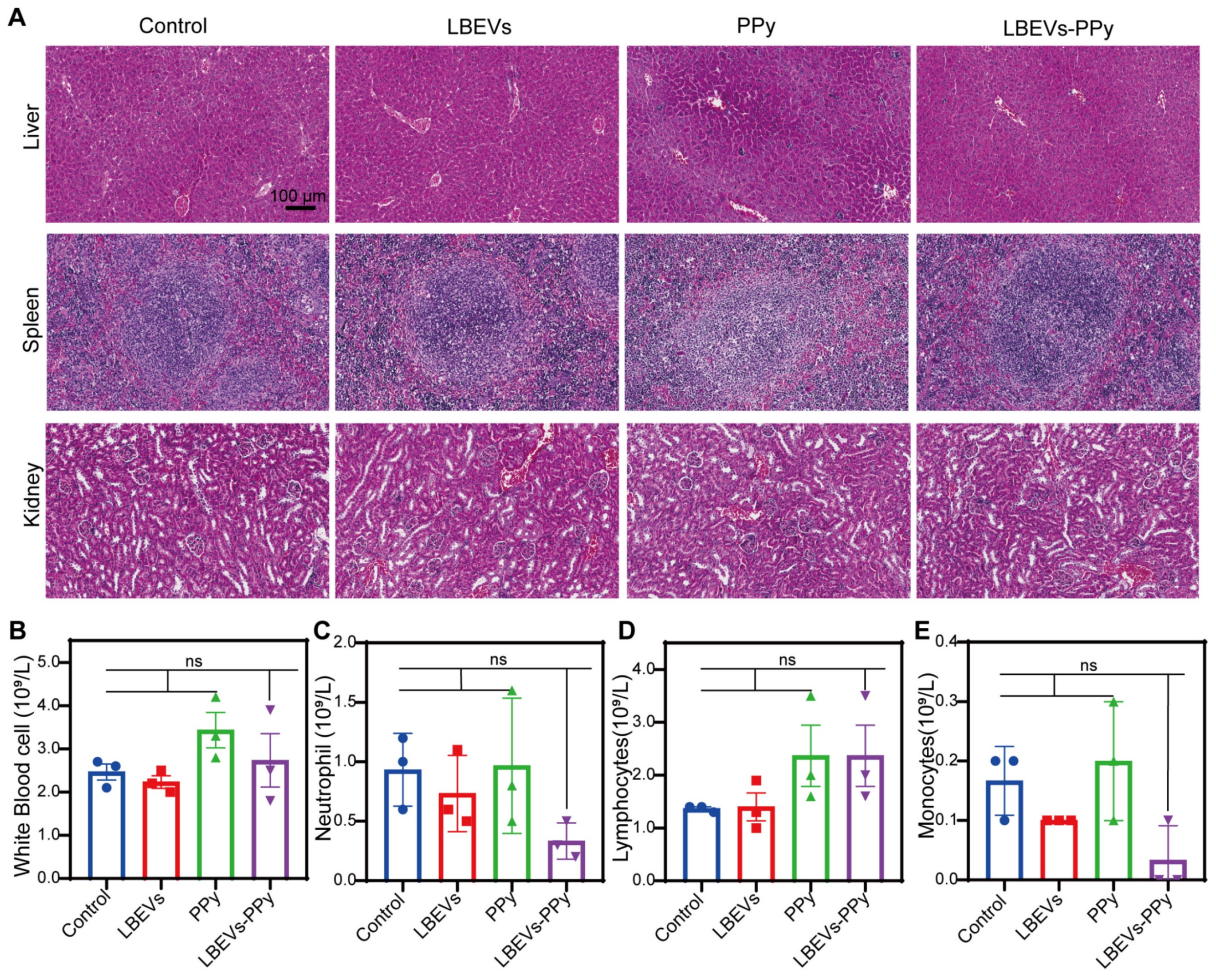
The safety assessment of LBEVs-PPy *in vivo*. (A) Changes in histomorphology were evaluated in different tissues with H&E staining. Scale bar: 100 μm. (B-E) Blood routine examination indexes (White Blood cell, Neutrophils, Lymphocytes, and Monocytes) of mice after different treatments. Sample size n = 3 for all experiments by a one-way ANOVA. Data are presented as means ± SEM. Ns: no significance.

To further evaluate the safety of LBEVs-PPy treatment *in vivo*, serum and pathological analyses related to biosafety were performed. As demonstrated by the H&E staining of vital organs in Figure 7A, compared with control mice, LBEVs-PPy-treated mice did not exhibit notable histological changes and cell destruction in the liver, spleen, and kidney. In addition, we collected orbital venous blood from mice for analysis on day 13. In LBEVs-PPy treatment group, there were no significant variations in blood routine indices, including white blood cell count, neutrophil count, lymphocyte count, and monocyte count (Figure 7B-E).

## Conclusion

In summary, we have successfully developed an *in-situ* spray-polymerized coating initiated by BEVs with NIR photothermal responsiveness for synergistic antibacterial treatment and accelerated wound healing. LBEVs, derived from probiotic *L.reuteri* that suppresses pathogens, are extracted and purified before surface functionalization by Fe^3+^ through electrostatic adsorption. LBEVs-PPy coatings are fabricated by co-spraying Fe^3+^ -functionalized LBEVs and pyrrole monomers onto wounds, which then triggers *in-situ* oxidative polymerization. By virtue of probiotic properties of parent bacteria, LBEVs-PPy coatings demonstrate excellent biocompatibility alongside potent efficacy against types of pathogens. Simultaneously, the incorporation of PPy endows the photothermal effects to upregulate *VEGFA* and *ANGPT1* expression in HUVECs, thereby enhancing angiogenesis. We finally validated that the LBEVs-PPy coating significantly accelerated wound healing through dual synergistic mechanisms-pathogen clearance by LBEVs and tissue regeneration potentiated by PPy's photothermal conversion in the mouse model of skin wound and infection. We anticipate that this work will provide a promising biotherapeutic strategy for infected wound treatment and tissue repair.

## Supplementary Material

Supplementary figures.

## Data Availability

The original data supporting the findings of this study (including *in vivo* and vitro experiments) are available from the corresponding author (Z.G.) upon reasonable request.

## Abbreviations

BEVs: Bacterial extracellular vesicles
LBEVs: *Lactobacillus reuteri*-derived BEVs
NIR: Near-infrared
PPy: Polypyrrole
LBEVs-PPy: Polypyrrole coating initiated by LBEVs
*VEGFA*: Vascular endothelial growth factor A
*ANGPT1*: Angiopoietin-1
HSP90: Heat shock protein 90
p-eNOS: Phosphorylated endothelial nitric oxide synthase
HUVECs: Human umbilical vein endothelial cells
MRS: de Man, Rogosa, Sharpe (medium)
DLS: Dynamic light scattering
BCA: Bicinchoninic acid (assay)
TEM: Transmission electron microscopy
LSCM: Laser scanning confocal microscopy
*S. aureus*: *Staphylococcus aureus*
TSB: Tryptic soy broth
CFU: Colony-forming units
*E. coli*: *Escherichia coli*
*S. epidermidis*: *Staphylococcus epidermidis*
SEM: Scanning electron microscopy
DMEM: Dulbecco's Modified Eagle Medium
HaCaT: Human immortalized keratinocyte cell line
NIH3T3: Mouse embryonic fibroblast cell line
FT-IR: Fourier transform infrared spectroscopy
qPCR: Quantitative real-time polymerase chain reaction
WB: Western blot
H&E: Hematoxylin and eosin (staining)
IF: Immunofluorescence
CD31: Platelet endothelial cell adhesion molecule
SD: Standard deviation
NTA: Nanoparticle tracking analysis
LC-MS: Liquid chromatography-mass spectrometry
3-HPA: Reuterin
ROS: Reactive oxygen species
DCFH-DA: 2',7'-Dichlorodihydrofluorescein diacetate
ANOVA: Analysis of variance
