# Supplementary material for: Bacterial vesicles-initiated in-situ spray-polymerized coating enables synergistic antibacterial-photothermal functionality for accelerating wound healing
Source: Theranostics. 2026 Feb 11;16(9):4566–79. doi: 10.7150/thno.126844 (PMC12964136; doi:10.7150/thno.126844)
Supplement: Supplementary file 1 — Supplementary figures. [file thnov16p4566s1.pdf]

# Supporting Information

Bacterial Vesicles-Initiated *In-Situ* Spray-Polymerized Coating Enables Synergistic Antibacterial-Photothermal Functionality for Accelerating wound healing

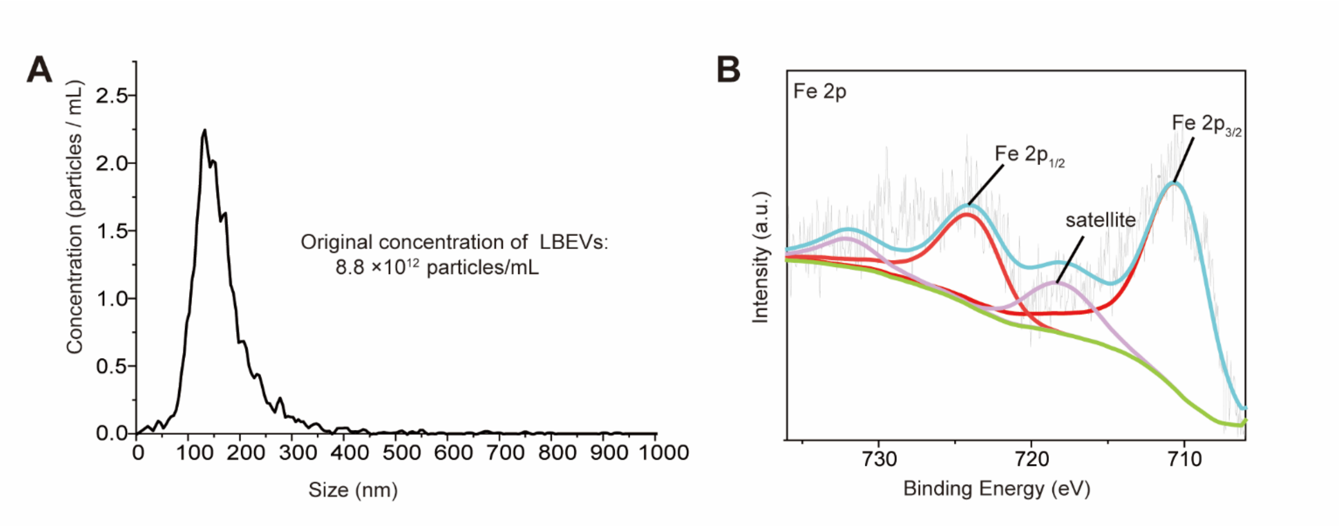


**Figure S1**. (A) NTA curve of concentrated LBEVs. (B) High-resolution XPS Fe 2p spectrum of the LBEVS-$Fe^{3+}$ sample.

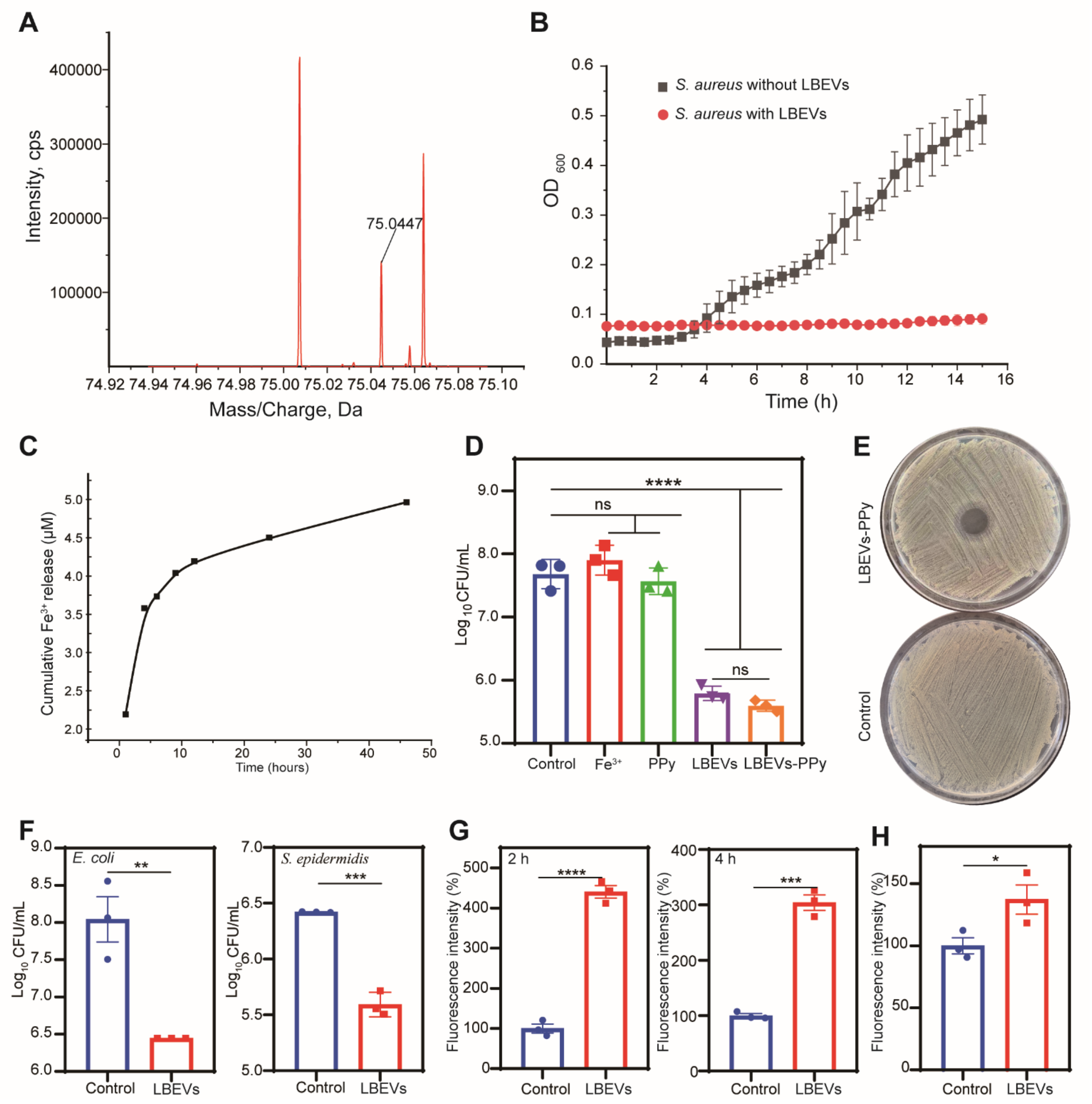


**Figure S2**. (A) Representative LC-MS plot of LBEVs. (B) Antibacterial effect of LBEVs assessed by bacterial growth curves. Growth of *S. aureus* was monitored by measuring OD $_{600}$ over 16 hours in TSB medium with or without the addition of LBEVs. (C) Kinetics of $Fe^{3+}$ release from the LBEVs-$Fe^{3+}$. (D) Quantitative comparison of the antibacterial efficacy of five groups (Control, $Fe^{3+}$, PPy, LBEVs, and LBEVs-PPy) against *S. aureus* after 24 hours of exposure. The viable bacteria were determined by CFU counts. Data are presented as $log_{10}$(CFU/mL). ****$p < 0.001$, ns: no significance. (E) Qualitative assessment of the antibacterial activity of LBEVs-PPy against *S. aureus* using the agar diffusion assay method. (F) Quantitative comparison of the antibacterial efficacy of LBEVs against *E. coli* and *S. epidermidis*. (G) The ROS levels in *E. coli* after treatment with LBEVs for 2 hours and 4 hours. (H) The ROS levels in *S. aureus* after treatment with LBEVs for 4 hours.

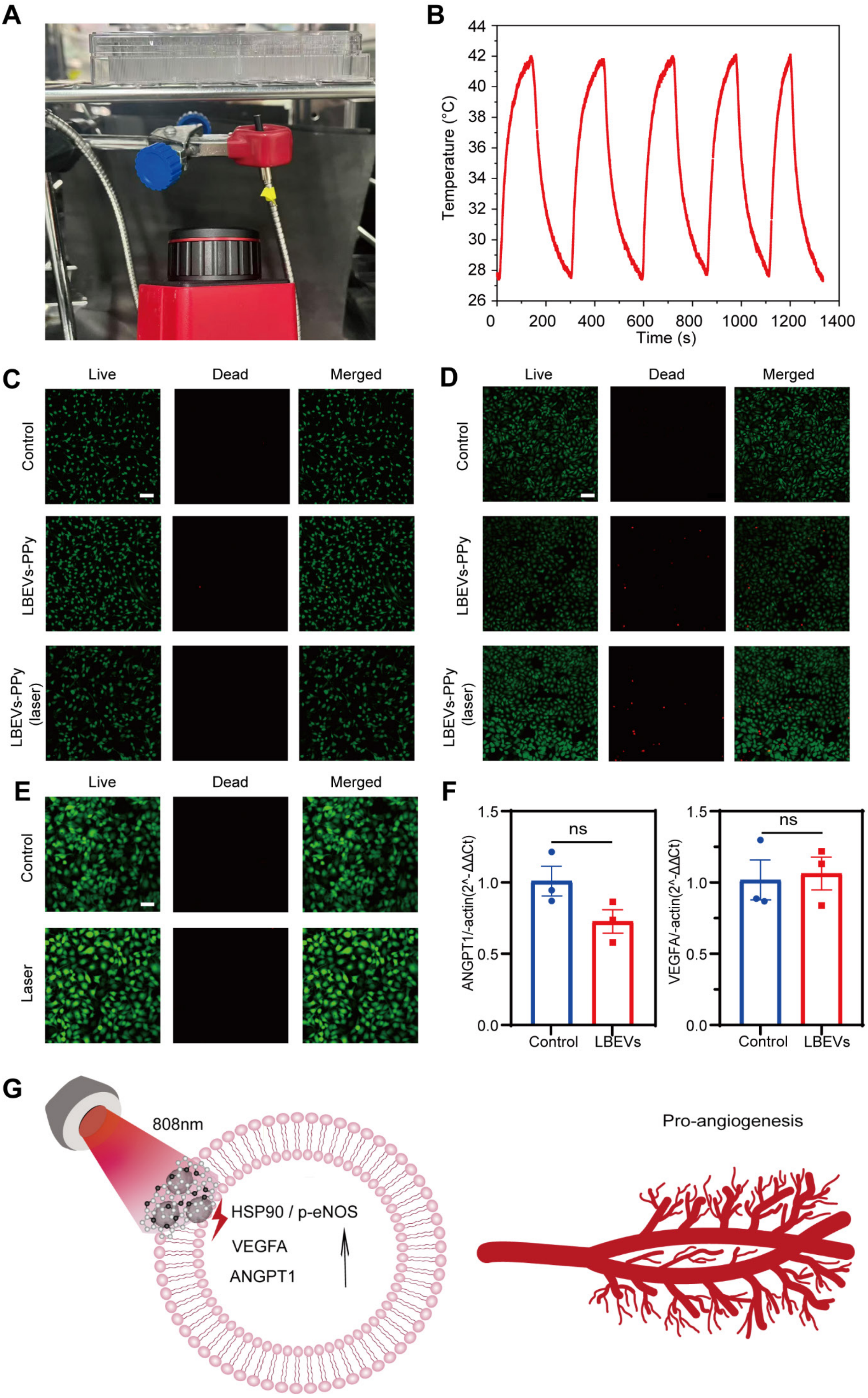
A
B
44
42
40
38
36
34
32
30
28
26
Temperature (°C)
0
200
400
600
800
1000
1200
1400
Time (s)
C
Live
Dead
Merged
Control
LBEVs-PPy
LBEVs-PPy (laser)
D
Live
Dead
Merged
Control
LBEVs-PPy
LBEVs-PPy (laser)
E
Live
Dead
Merged
Control
Laser
F
1.5
1.0
0.5
0.0
ns
ANGPT1/-actin(2^-ΔΔCt)
Control
LBEVs
VEGFA/-actin(2^-ΔΔCt)
G
808nm
HSP90 / p-eNOS
VEGFA
ANGPT1
Pro-angiogenesis

**Figure S3.** (A) Schematic diagram of the experimental setup for *in vitro* photothermal performance evaluation. (B) Heating curve of LBEVs-PPy for five on/off cycles at a power density of 0.8 W/cm². (C): Cell live/dead staining fluorescence images of NIH3T3. Scale bar: 100 µm. (D) Cell live/dead staining fluorescence images of HaCAT. Scale bar: 100 µm. (E) Representative live/dead fluorescence images of HUVECs following 808 nm, 0.8 W/cm² laser irradiation. Scale bar: 100 µm. (F) The relative mRNA expression levels of pro-angiogenic genes VEGFA and ANGPT1 in HUVECs after co-incubation with LBEVs. ns: no significance. (G) The possible mechanisms of thermal stimulation stimulates angiogenesis.

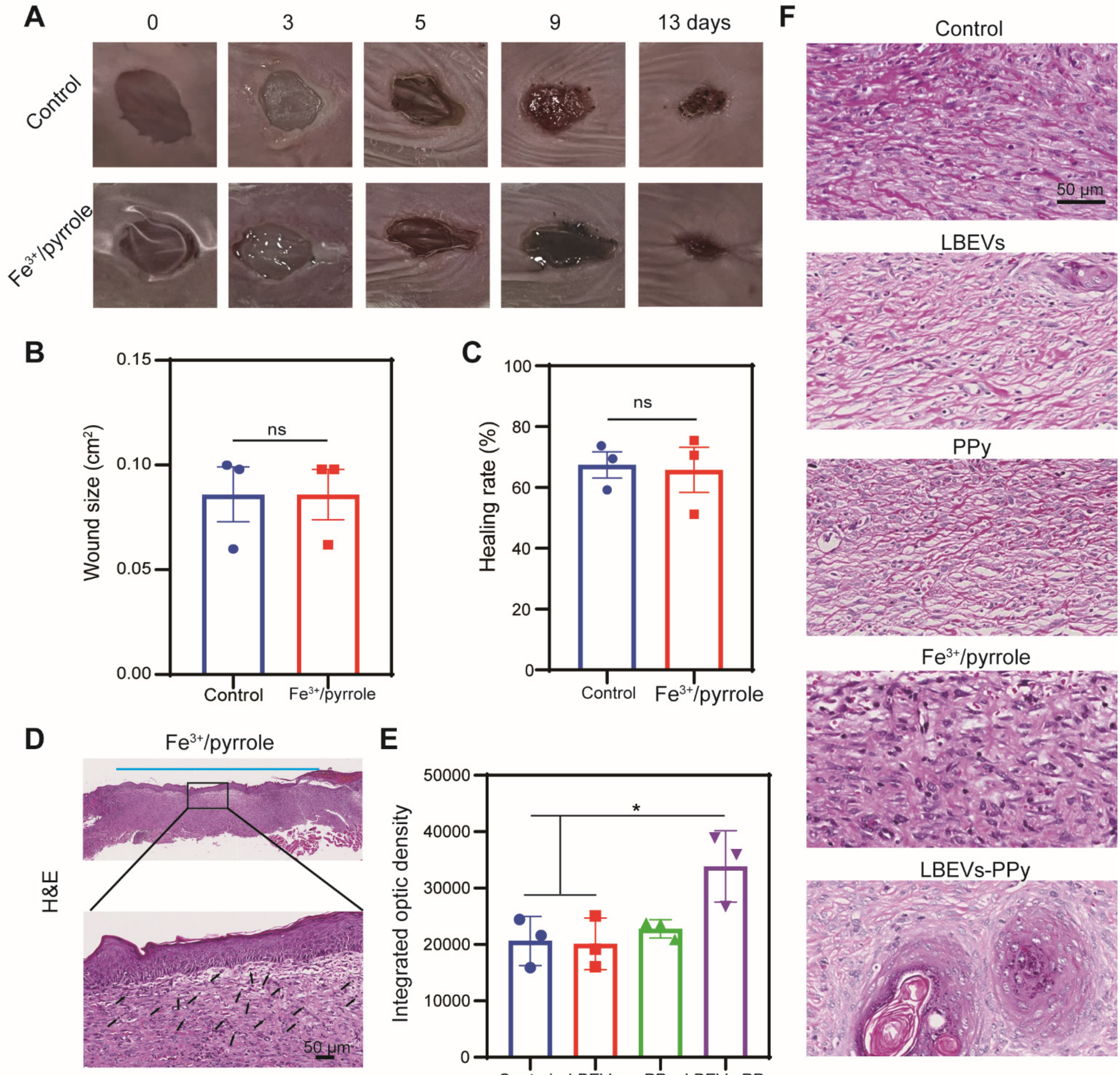


**Figure S4.** (A) Representative wound appearances in the control and $Fe^{3+}$/pyrrole groups at postoperative days 0, 3, 5, 9, and 13. Quantification of (B) wound closure size and (C) wound healing rate of control and $Fe^{3+}$/pyrrole groups during treatment. Ns: no significance. (D): Representative H&E staining images of wound tissues from $Fe^{3+}$/pyrrole group on day 13. Scale bar: 50 µm. (E) Quantitative analysis of VEGF expression in wound tissues. *$p < 0.05$. (F): Representative high-magnification H&E staining images of wound tissues from each group. Scale bar: 50 µm.
